# Development of Quality Control Methods for Dispersibility and Stability of Single-Wall Carbon Nanotubes in an Aqueous Medium

**DOI:** 10.3390/nano11102618

**Published:** 2021-10-05

**Authors:** Moran Ben Basat, Noa Lachman

**Affiliations:** 1 Department of Materials Science and Engineering, Faculty of Engineering, Tel-Aviv University, Ramat Aviv, Tel Aviv 6997801, Israel; moran@nemonano.com; 2 Nemo Nanomaterials Ltd., Petah-Tikva 4951025, Israel

**Keywords:** single walled carbon nanotubes (SWCNT), electrical properties, dispersion quality

## Abstract

The attractive properties of single-wall carbon nanotubes (SWCNT) such as mechanical strength and high electrical and thermal conductivity are often undercut by their agglomeration and re-agglomeration tendencies. As a result, the application of SWCNT as additives in advanced composite materials remain far from their potential, with proper dispersion being the major inhibitor. This work presents a dispersion quality control approach for water-based SWCNT dispersions (dispersed by a unique combination of physical and chemical methods), using complementary and easily scalable, characterization methods. UV-Vis spectroscopy, rheological measurements, and precipitant sheet resistance were used to understand the properties of the initial solution through processing and application. From an industrial perspective, these methods are fast and easy to measure while giving a repetitive and quick indication of dispersion quality and stability. The methods were correlated with microscopy and Raman spectroscopy to validate dispersion and SWCNT quality under various dispersing energies. The protocol was then applied to estimate the stability of SWCNT solutions, as well as the effectiveness of different surfactants in aiding dispersion. The simple, fast, and scalable combination of different characterizations provides good SWCNT dispersion and can be used as a quality control system for industrial production and usage.

## 1. Introduction

Carbon nanotubes (CNT) in general, and single walled carbon nanotubes (SWCNT) in particular, are known for their properties of high mechanical strength, electrical conductivity, and thermal conductivity [1]. Such properties make SWCNT ideal for various commercial applications such as solar panels [2], hydrogen storage [3], conductive inks for flexible displays [4], low-weight conductive reinforcements in polymers [5], electrochemical active materials for supercapacitors [6], biofuel cells [7], adhesive materials [8], and more. To harness these properties, SWCNTs often need to be integrated into a medium (either solid or liquid) and should be individually dispersed. Nevertheless, high quality stable dispersion still presents a challenge, as the high surface area of SWCNT makes them susceptible to aggregation, driven by strong Van der Waals forces [1,9].

Therefore, great efforts are invested in achieving the proper dispersion of SWCNT on an industrial scale. The quality assessment of such efforts includes methods of characterizing stability and dispersibility in liquid media such as electron microscopy [10,11], UV-Vis and NIR spectroscopies [9,12,13,14,15], rheology [16,17,18], and Raman spectroscopy [19]. UV-Vis-NIR absorbance spectroscopy can be used both as an indicator for dispersion quality and as a quantitative method to estimate the SWCNT concentration in the solution [13,20]. Rheological measurements characterize the solution’s shear thinning behavior and the relationships between the storage modulus (G′) and the loss modulus (G″), with reports correlating prominent shear thinning to significant aggregation [21]. Conductivity can also be used as an indicator, as well-dispersed SWCNT are reported to be up to 50 times more conductive than their aggregated form [22].

However, all of these aforementioned methods were validated separately, mostly by adhering to visual methods such as UV-Vis spectroscopy and electron microscopy. Though highly accurate, electron microscopy is expensive and time-consuming, making it unfit for large-scale quantitative characterization. In this work, we correlate UV-Vis, rheology, and surface conductivity to each other throughout the dispersion process, gaining a better understanding of SWCNT dispersibility on an industrial scale. Moreover, we acquired an increased qualitative accuracy of dispersion characterization and assessed its implications both on processing and on the final product characteristics.

## 2. Materials and Methods

**Figure 1:**
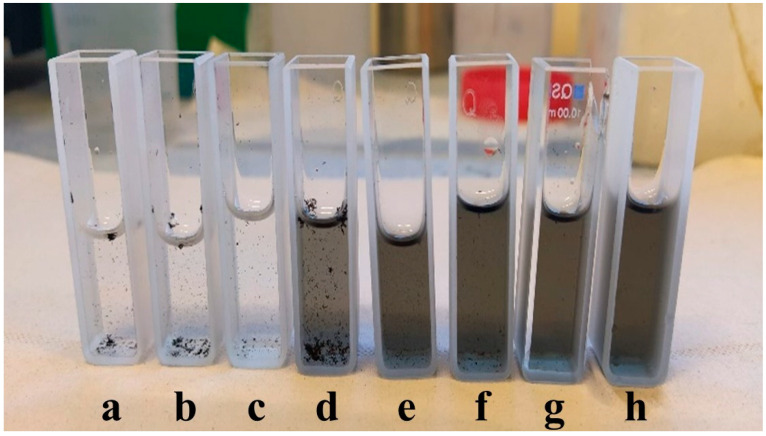
SWCNT dispersion samples taken from the same batch during the dispersion process after different energy invested: (**a**) 27 Wh; (**b**) 55 Wh; (**c**) 82 Wh; (**d**) 109 Wh; (**e**) 136 Wh; (**f**) 164 Wh; (**g**) 191 Wh; and (**h**) 218 Wh, shown here in the standard quartz cuvettes used for UV-Vis spectroscopy.

SWCNT, at 2 mg/mL, (Tuball powders, purchased from OCSiAl (Novosibirsk, Russia)), cat. numbers TUBALL SWCNT 01RW02 with a length of more than 5 µm and 85% purity according to the manufacturer data sheet) were dispersed with anionic aromatic surfactants in distilled water (type III) using the Nemo Nanomaterials Ltd. (Petach Tiqva, Israel) proprietary process, based on a unique combination of mixing and dispersing methods applying physical shear forces, formation and collapse of low-pressure bubbles in liquids, along with a special combination of different dispersing agents (Nemo Nanomaterials Ltd. lot number D3-14-1). During the process, samples from the same batch were taken at constant time intervals corresponding to different energy points calculated by the power supply for further characterization (see Figure 1). Energy was used as a reference variable to simplify the comparison between the current dispersion method and other well-known methods of dispersion in liquid volume such as ball-milling and high-shear mixing and to eliminate the results’ dependency on the machine setup. A final sample was taken out after more than twice the standard processing time to test the effect of extended processing. It should be noted that no filtering or other precipitant separation was used, as a simulation of industrial processes where the solution will be used “as-is”.

Optical microscope: SWCNT dispersions were placed on a glass slide and measured under an optical microscope (Eclipse LV100ND by Nikon, Tokyo, Japan, 20× magnification, bright field) to monitor dispersion progress and dispersibility.

High resolution transmission electron microscope (HR-TEM): Each SWCNT dispersion was placed on a 400-mesh copper grid with ultrathin support carbon film, 3 nm thick. The grids were dried in the open air and scanned with an ultrahigh-resolution transmission electron microscope (JEOL™ JEM-2010F UHRFEG-STEM, CCD US1000 camera manufactured by Gatan (Pleasanton, CA, USA), resolution 2 K × 2 K for image recording, 200 KV voltage, parallel illumination, and bright field conditions) to evaluate dispersion level, diameters, and tube lengths.

Raman spectroscopy: SWCNT dispersions were placed on glass slides and dried in the open air. The slides were measured by LabRAM HR Evolution, Horiba (Piscataway, NJ, USA), with a wavelength of 532 nm (green laser) in a region 1000–3000 cm^−1^ for I_D_/I_G_ ratio calculation. All the measurements were performed at room temperature.

Spectrophotometer: The absorbance of 2 mg/mL water-based SWCNT dispersion was monitored at different process times (related to different processing energies and correlated with different dispersion levels of the CNT). All samples were diluted and mixed in a 1:100 ratio with DI water (type III). The absorbance was measured in a range of 190–900 nm by JASCO V750. The absorbance of the reference sample, which included DI water and all other dispersion components in the same concentrations as the sample except for the CNT (“matrix absorbance”), was subtracted from the tested sample. A plot of absorbance vs. wavelength was created for each of the tests. The spectrophotometer program calculated the resonance ratio and spectral width.

Rheometer: Rheology parameters were tested by a Thermo Scientific™ HAAKE™ MARS60™ rheometer (Waltham, MA, USA) with a cone (60 mm, 1°) and plate geometry, while maintaining a 25 °C temperature, in two different programs: shear rate to shear stress plot with constant shear stress and shear rate range from 0.1 to 4300 1/s; amplitude sweep (with constant frequency ω = 10 rad) for estimation of G′, G″ relationships and LVR region in a range of 0.01–50 shear strain (ϒ).

Sheet resistance meter: PET film (125 µm, Jolybar) was coated with a 12 µm (wet thickness) sample from each of the dispersions by a rod coating tool with 12 µm calibrating rod to ensure a uniform coating. All samples were dried at 150 °C for 2 min inside an oven and tested by a resistivity meter (PORTABLE), with precision resistance measurements from 0.1 Ω to 2.0 × 10^14^ Ω, using a PRF-911 Concentric Ring Set to obtain the surface resistivity results by ASTM D-257. To convert surface resistance to surface resistivity, the results were multiplied by 10 (geometry correction factor). Each of the PET films was measured in 5 different places (top, center, bottom, and two sides of the film page) and the average values are presented below.

Dispersion stability: Dispersion stability over time (for hand-mixed dispersions to guarantee homogenization, equivalent to 218 Wh sample) was tested for 28 days at 7 different points: 1, 2, 3, 7, 14, 21, and 28 days. Dispersion optical absorbance, sheet resistance, and rheological properties were characterized each time and normalized to t = 1 day values (one overnight after the process was complete).

Surfactant efficiency: 6 different types of surfactants were used to disperse 2 mg/mL SWCNT in water. All the samples were dispersed by the same process but differed from each other in the energy required to reach full homogenous dispersion. The surfactants used were anionic aromatic (Lot D3-14-1) and anionic aliphatic (Lot D3-175-2) to investigate the benzene ring effect on surfactant–CNT bonding, cationic (Lot D3-66-1) to examine the headgroup charge effect compared with the anionic surfactants, graft polymer (Lot D3-59-1), LMW polymer (average Mw ~90,000, Lot D3-61-2), and HMW polymer (average Mw ~250,000, 2.7-fold longer than LMW, Lot D3-68-2) to examine the surfactant structure and molecule size effects on dispersibility.

## 3. Results and Discussion

### 3.1. Spectroscopic Analysis

**Figure 2:**
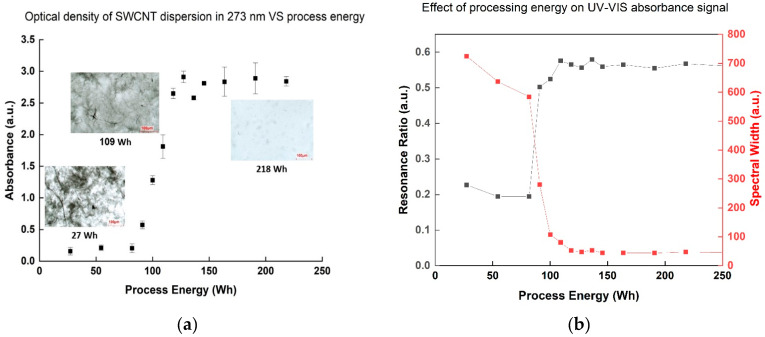
(**a**) Optical absorbance density of SWCNT dispersion samples in 273 nm dependent on the process energy. Optical microscopy images are presented for 3 process points: 27 Wh (beginning of the process), 109 Wh (middle), and 218 Wh (end of process). The scale bar in the images is 100 µm. (**b**) Resonance ratio and spectral width of the 273 nm peak dependent on the process energy.

The absorbance intensity of SWCNT dispersion samples at the 273 nm wavelength [14], corresponding to a signature of the surface π-plasmon excitation of SWCNT [22], showed an increasing trend during the process, indicating improved dispersion—up to a point. Specifically, the absorbance increased dramatically after employment of 109 Wh, but any additional energy investment above 145 Wh showed a negligible effect (Figure 2a). The resonance ratio (peak area normalized to background area at the same range) and the normal spectral width (peak width at half height divided by its height) are two quantitative characteristics of the absorbance peak that can indicate suspension quality [23,24]: when the normalized spectral width decreases and the resonance ratio increases, the absorbance band shape is sharper and more intense, indicating an increase in the number of suspended individual nanotubes. As both are quantitative parameters, comparison between different dispersions becomes easier, which can be an advantage in terms of quality control method. Indeed, both parameters showed similar conclusions (Figure 2b): at 109 Wh, the resonance ratio increased dramatically, and the normal width dropped, with little change resulting from further energy investment. These results also correlated with the optical microscopy observation showing the most significant change in dispersion contrast at 109 Wh, followed by negligible improvement when investing more than 136 Wh. These findings help determine a stop-point for dispersion processing, as excessive energy investment results in defect creation on the SWCNT themselves [24,25]. Such damage can be further confirmed by the increase in the I_D_/I_G_ ratio of the Raman spectra (Table 1), often used to characterize defects in CNTs [26], in direct relation to the invested energy.

### 3.2. HR-TEM

**Figure 3:**
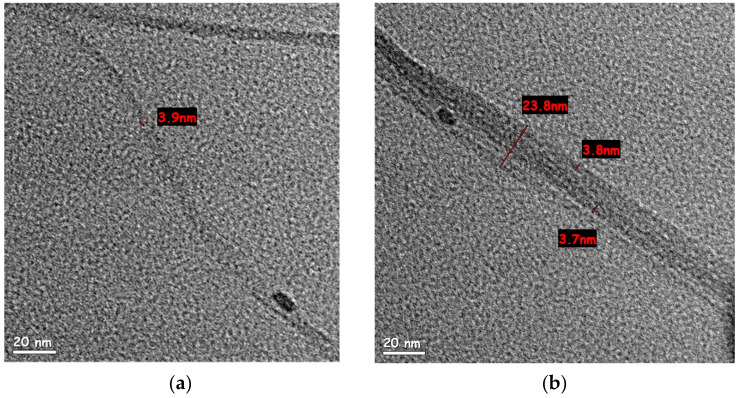

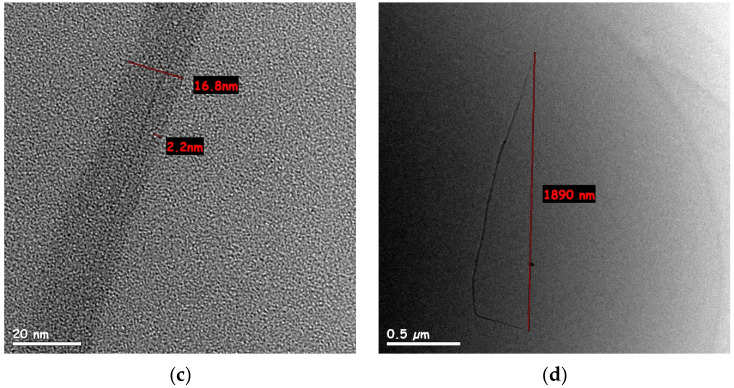
HR-TEM images of different bundles size for SWCNT in water-based dispersion after 218 Wh: (**a**) bundle split, one of the sections is of 3.9 nm diameter, containing 2–3 tubes (scale bar 20 nm); (**b**) 23.8 nm bundle (scale bar 20 nm); (**c**) 16.8 nm SWCNT bundle after 600 Wh dispersion (scale bar 20 nm); (**d**) a low magnification TEM image of SWCNT bundle after 600 Wh dispersion. Its length, measured edge to edge, was approx. 2 µm (scale bar 0.5 µm).

SWCNT water-based dispersions were examined under high-resolution TEM, to observe the effect of high-energy dispersion (218 Wh and 600 Wh, respectively) on the tube diameter and separation within bundles. According to the SWCNT manufacturer, the raw tubes have a diameter of 1.6 ± 0.5 nm and a length of more than 5 µm. The dispersion in 218 Wh (Figure 3a,b) showed a variety of bundles with an average diameter (measured over 10 bundles) of 20.9 ± 5 nm. SWCNT dispersion after 600 Wh (Figure 3c) showed significantly thinner bundles (average diameter of 13.5 ± 2.5 nm measured over 10 bundles), supposedly indicating better dispersion. However, while measuring the length of an individual tube is not trivial, bundles observed after 600 Wh exhibited a length of ~2.2 µm (Figure 3d)—shorter than the reported length of an individual tube. This observation, combined with Raman spectroscopy, indicated an increase in SWCNT defects and fracture after 600 Wh.

It should be noted that as an industrial characterization method, TEM is not only expensive and time-consuming but also very limited in its scope. The images presented here were chosen to reflect as much as possible on the average values. A different choices of images could show, for example, bundles after 218 Wh, which were closer to the average values of bundles after 600 Wh. In contrast, methods as UV-Vis, shear-rate viscosity, and sheet resistance take only a few minutes to run, thus allowing for real-time status assessment throughout production. Furthermore, the quick measurements allow statistical analysis within the same batch, thus increasing statistical reliability. If industrial production of SWCNT is to be practical, reliable quality analysis of kilograms per day is required. The narrow frame of TEM—the benchmark of dispersion analysis in lab scale research—cannot answer such a demand.

### 3.3. Rheological Properties

**Figure 4:**
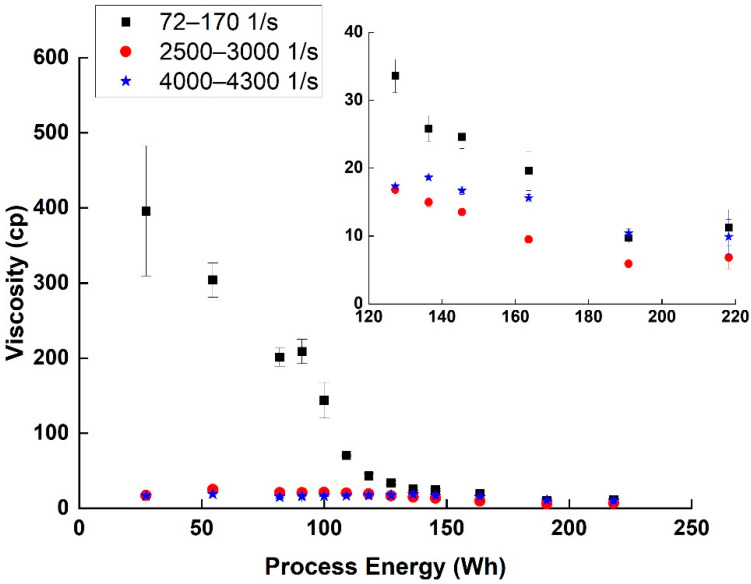
Viscosity averages and SD values (η in cp) in 3 different shear rate zones for various SWCNT dispersion energies. Inset is a zoom-in on the 120–220 Wh area.

As can be seen (Figure 4), the shear-thinning effect, which is common in suspensions [17] decreased with the advancement of the dispersion process (i.e., increasing the invested energy). The plateau region (2500–3000 1/s) viscosity measured also decreased with the dispersion process from 25 cp at 54 Wh to 7 cp at 218 Wh. This decrease in shear-thinning and viscosity is usually connected to a decrease in the size of agglomerates resisting the flow, and thus is used as an indication of better dispersion [17,18].

By performing the amplitude sweep test, and by comparing the linear viscoelastic region (LVR), G′ and G″, the SWCNT network structure and dispersion viscoelasticity behavior under different energy investments can be better understood. In the initial process steps (up to 164 Wh), the dominant behavior was viscoelastic: initially G′ was greater than G″, and the dispersion showed a gel-like structure (Table 2). After the crossover shear strain value, the relation between G′ and G″ was changed, the network structure was broken, and the dispersion behaved like a liquid. Both G′ and G″ absolute values decreased with the dispersion energy, indicating again decreased elasticity behavior. At higher processes energy (190 Wh and above), G″ became dominant in all shear strain range, indicating liquid behavior and high dispersion level of the SWCNT compared to the previous steps.

### 3.4. Sheet Resistance

**Figure 5:**
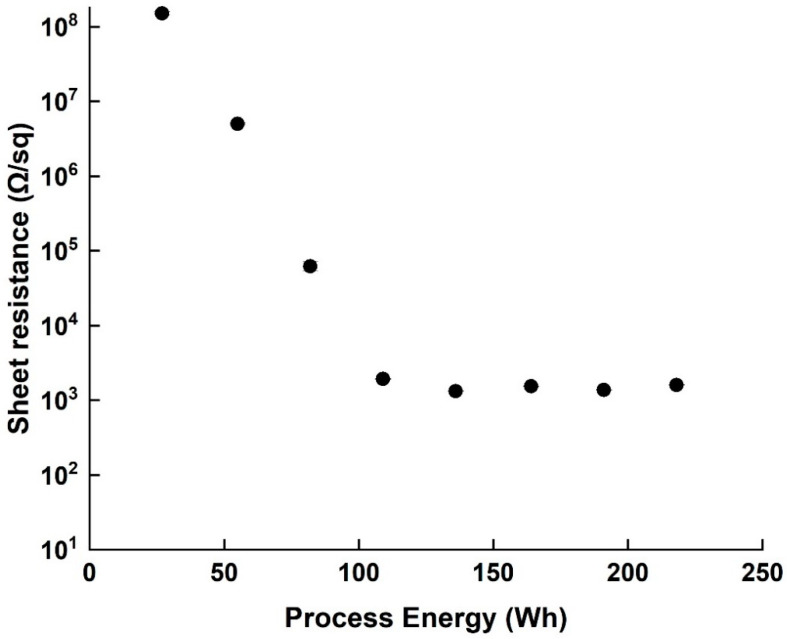
Sheet resistance values of PET film coated with dispersion after different process energy points (27–218 Wh).

The sheet resistance of all samples was tested as a characterization property, and the resistivity values were recorded during the process (Figure 5). At the beginning of the dispersion (27–82 Wh) the coating uniformity of the SWCNT on the PET film was poor, displaying many agglomerates, and a relatively high sheet resistance of 10^8^ Ω/sq. The sheet resistance decreased with increased invested energy up to 109 Wh, but no changes in resistance magnitude were observed between 109 Wh and 218 Wh. It should be noted that over-processing (600 Wh) increased the sheet resistance of the film by one order of magnitude (from 10^3^ Ω to 10^4^ Ω), most likely due to the fact established by HR-TEM and Raman spectroscopy that the SWCNT themselves are damaged. It is also worth mentioning that the standard deviations of measured resistance decreased with the resistance itself (from 8.34 × 10^6^ to 8.9, both smaller than the point marks)—another indication of the improved dispersion.

### 3.5. Application #1: Determination of Dispersion Stability

**Figure 6:**
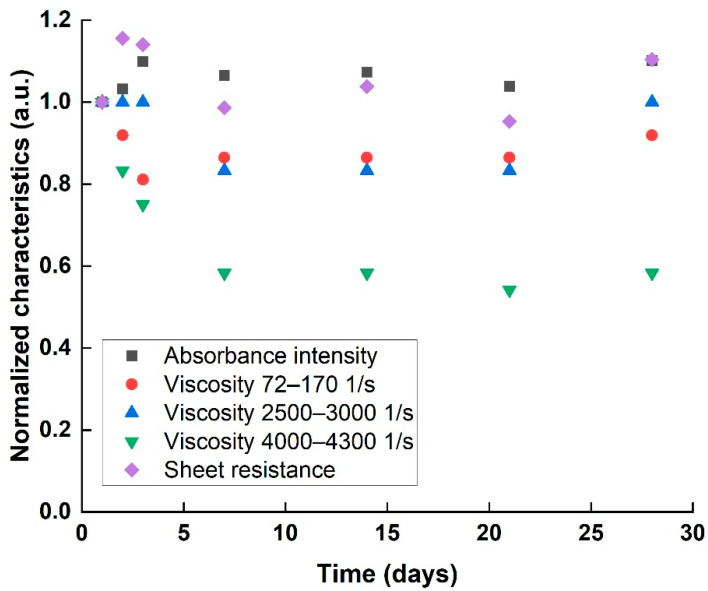
Absorbance intensity, viscosity in various shear rates, and sheet resistance values of aging 218 Wh dispersion process, normalized to 1st day values.

The stability of a homogenous dispersion (218 Wh sample) was examined over 28 days, according to the proposed characteristics. Optical absorbance, rheological properties, and sheet resistance were characterized each time and normalized to the first day values (Figure 6). The optical absorbance and sheet resistance results indicated a stable dispersion for 28 days, with negligible changes in characteristics over the measurement time. However, normalized viscosity exhibited a decreasing trend over time. Such a decrease in viscosity can hint at the beginning of agglomeration process, either a decreasing particle specific surface area that causes a decrease in the internal friction to the fluid’s flow, or by decreasing the overall concentration by particle precipitation. As the re-agglomeration is only at its initial stage, the effect is too small to be observed either optically or electrically, indicated only by the far more sensitive rheology results.

### 3.6. Application #2: Optimizing Surfactant

**Figure 7:**
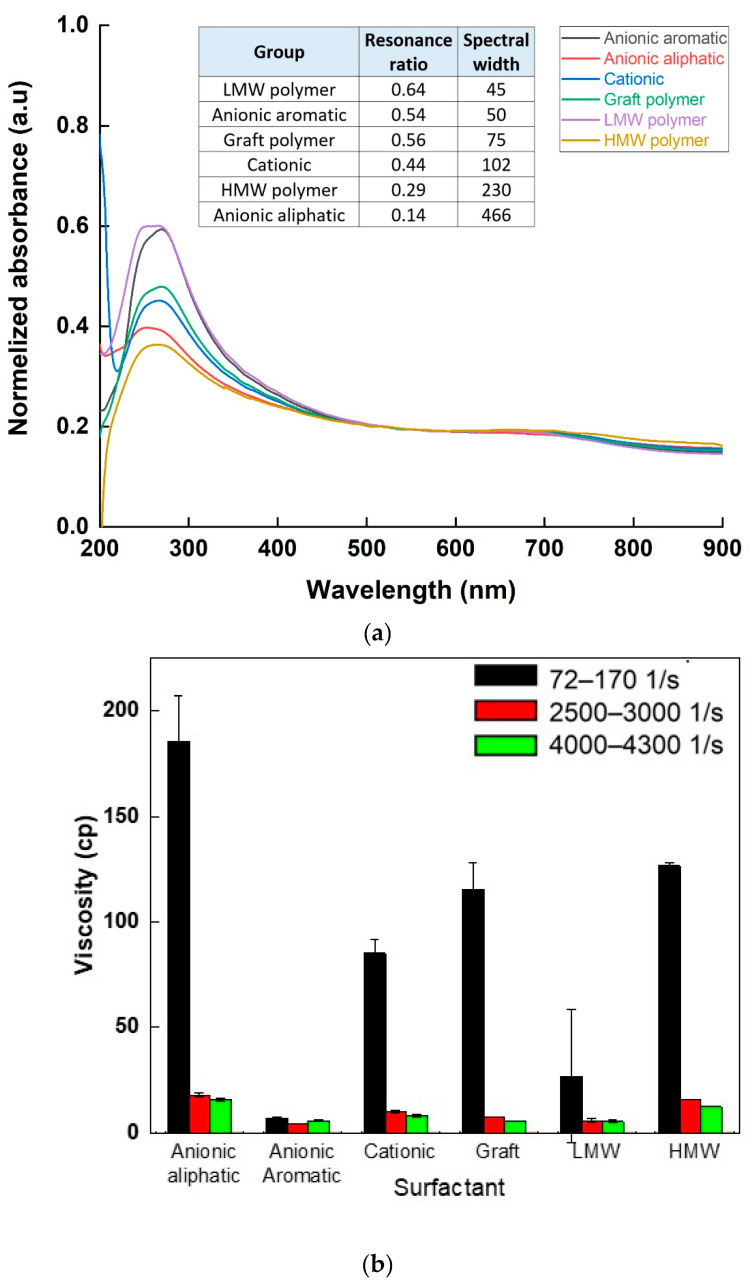

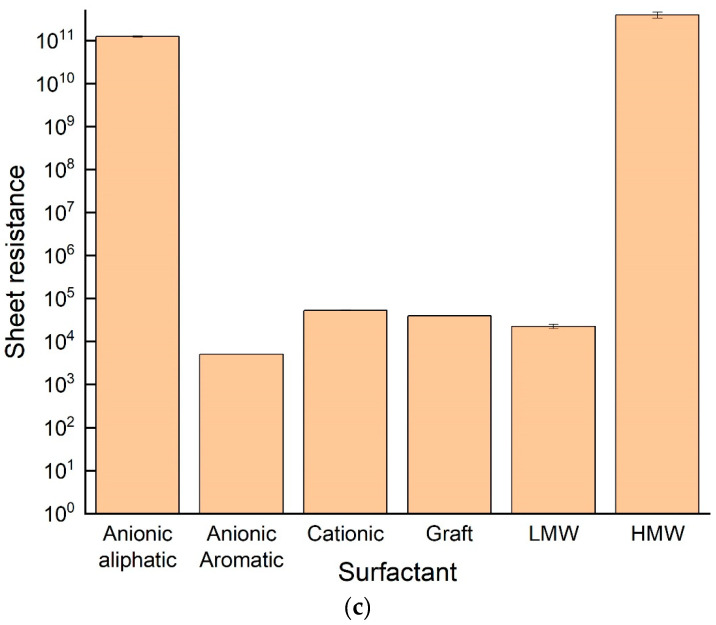
Absorbance intensity (**a**), viscosity in various shear rates (**b**), and sheet resistance (**c**) values of SWCNT suspensions dispersed in various surfactants.

A total of 6 different types of surfactants were used to create SWCNT aqueous dispersions, and the resultant dispersions were analyzed according to the proposed characteristics: optical absorbance, rheological properties, and sheet resistance were characterized for each surfactant type. The UV-Vis absorbance spectrum (Figure 7a), normalized to the same baseline, indicated a difference both in peak height and sharpness between different surfactants: the highest absorbance values were observed when using anionic aromatic and LMW polymer surfactants, while the lowest one was observed using HMW polymer. A similar trend was observed quantitatively, comparing the resonance ratio and spectral width of the spectra (inset table, Figure 7a). Rheological behavior (Figure 7b) was comparable: although shear-thinning behavior in low shear rate was seen in all dispersions, the level of shear-thinning, which is related to SWCNT network creation, was different between surfactants. Both anionic aromatic and LMW polymer surfactants exhibited lower initial viscosity and lower shear-thinning than all the other surfactants. The anionic surfactants also had a very low G′ to G″ crossover point in the amplitude sweep (Table 3), showing a “liquid-like” behavior relating to low SWCNT agglomerates percentage and good dispersibility. Sheet resistance (Figure 7c) complimented the characteristics, with HMW polymer and anionic aliphatic surfactants showing the highest sheet resistivity values (~10^11^ Ω/sq), and anionic aromatic demonstrating the lowest resistivity (~10^3^ Ω/sq). Clearly preferred surfactants emerged from all three characteristics: the anionic aromatic, with the LMW polymer as a close second. These results demonstrate the importance of surfactants as dispersion agents: processability (i.e., rheological properties) and final properties (e.g., electrical conductivity) are strongly affected not only by the properties and percentage of the filler but also by the quality of the dispersion. These results also propose “rules of thumb” for selecting an appropriate surfactant for SWCNT dispersibility: first and foremost, π-π stacking interactions [27], enabled by the aromaticity of the surfactant, stabilize the suspension better than electrostatic repulsion alone (seen by comparing aromatic to aliphatic anionic surfactants), most likely by strengthening the absorbance of the surfactant to the SWCNT. Moreover, as the surfactant should also stabilize the suspension sterically, small molecules penetrate the small spaces between the individual tubes in the agglomerates more easily [28,29], and exfoliate the tubes more efficiently, as demonstrated by the very clear advantage of the LMW polymer over its higher molecular weight counterpart as a surfactant.

## 4. Conclusions

In this study, different SWCNT water-based dispersions were prepared and characterized by three scalable methods—UV-Vis spectroscopy, rheological measurements, and sheet resistance—to estimate dispersion quality and stability. The characterization methods indicated the progress of dispersion process, showing different sensitivity to different stages. Optical density and sheet resistance plateau (~160 Wh) marked the end of suspension homogenizing, while rheological measurements—the most sensitive method—identified the breaking of networks (~220 Wh). Investing further energy in dispersion resulted in damage to the tubes, indicated by further increase in absorbance intensity and sheet resistance, as well as further decrease in viscosity. High-resolution transmission electron microscopy and Raman spectroscopy verified these observations. The aforementioned three methods were also used to characterize dispersion stability over time (28 days). The results showed a mostly stable dispersion in terms of optical and electrical properties, with the more sensitive rheology suggesting the beginning of precipitation. Lastly, the methods were used to examine the effect of different surfactants on the dispersion quality. All three methods identified the same surfactant as the most efficient surfactant for a SWCNT water-based system. This agreement between methods establishes the superiority of the chosen formulation in optical properties and visual impression, rheological properties during processing, and electrical properties of the final product. Therefore, this method combination allows for a fast and cheap formulation optimization compared to methods such as TEM (in terms of operation costs, operator skills, and extra equipment). All together, these three fast and easily scalable methods combined prove to be a simple and powerful quality control tool for SWCNT water-based dispersions, providing good prediction on homogeneity, processibility, and ultimate properties. Thus, the combined protocol can significantly lower the barrier of making and using SWCNT on an industrial scale, an important step in harnessing the potential of SWCNT in practice.

## Figures and Tables

**Table 1:** Intensity of G and D Raman peaks for 3 points along the SWCNT dispersion process and I_D_/I_G_ ratio calculation.

Process Energy (Wh)	I_D_	I_G_	I_D_/I_G_ Ratio
55	772	50,435	0.015
218	1558	59,973	0.026
600	1522	30,532	0.05

**Table 2:** LVR values and crossover point (Pa) for SWCNT dispersion samples.

Process Energy (Wh)	End of LVR (γ)	Crossover (Pa)	LVR Behavior
55	non	34.18	G′ > G″
82	0.03	17.37	G′ > G″
109	0.08	4.263	G′ > G″
136	0.16	1.966	G′ > G″
164	0.08	0.6324	G′ > G″
191	0.64	0.36	G′ > G″ but very close each other
218	0.52	Non	G″ > G′ for all γ range
245	0.46	Non	G″ > G′ for all γ range
273	0.35	Non	G″ > G′ for all γ range
600	non	Non	G″ > G′ for all γ range

**Table 3:** LVR values and crossover point (Pa) for SWCNT suspensions dispersed in various surfactants.

Surfactant Group	End of LVR (γ)	Crossover (Pa)	LVR Behavior
Anionic aromatic	non	0.2495 Pa	G″ > G′
Anionic aliphatic	non	52.46 Pa	G″ > G′
Cationic	0.008150	11.16 Pa	G′ > G″ Almost similar
Graft polymer	non	17.10 Pa	G′ > G″ in LVR
LMW polymer	0.06764	1.280 Pa	G′ > G″ in LVR
HMW polymer	non	55.59 Pa	G′ > G″ in LVR

## Data Availability

The data presented in this study are available on request from the corresponding author.
